# Deficiency of Self-Efficacy in Problem-Solving as a Contributory Factor in Family Instability: A Qualitative Study

**Published:** 2018-01

**Authors:** Zahra Pourmovahed, Seyed Saied Mazloomy Mahmoodabad, Hassan Zareei Mahmoodabadi, Hossein Tavangar, Seyed Mojtaba Yassini Ardekani, Ali Akbar Vaezi

**Affiliations:** 1 Department of Health Education and Promotion, Social Determinants of Health Research Center, School of Public Health, Shahid Sadoughi University of Medical Sciences, Yazd, Iran.; 2 Department of Education and Psychology, Yazd University, Yazd, Iran.; 3 Department of Nursing Education, Research Center for Nursing and Midwifery Care, Shahid Sadoughi University of Medical Sciences, Yazd, Iran.; 4 Department of Psychology, Research Center of Addiction and Behavioral Sciences, Shahid Sadoughi University of Medical Sciences, Yazd, Iran.

**Keywords:** *Efficacy*, *Family*, *Instability*, *McMaster Model*, *Problem*

## Abstract

**Objective: **Problem-solving ability is one of the most important means of family stability that enables the families to understand their roles, functions, and performances. Self-efficacy deficiency in problem-solving runs through many families. This qualitative study was conducted to investigate and describe how couples solve problems in their families.

**Method: **This study was conducted to detect couples’ self-efficacy deficiency in problem-solving using purposive sampling method. Several deep semi-structured interviews based on McMaster model and observations were conducted by nine family therapists and psychiatrists on four couples (eight persons) living in Yazd (Iran).The interviews were performed, audio-recorded, and transcribed verbatim. The analysis was interpreted through directed content analysis methods.

**Results:** Families in Yazd (Iran) made some attempts to solve their problems, but their efforts were not enough, and thus they suffered from self-efficacy deficiency, which included 8 categories. The main theme distilled from the data of 17 participants was self-efficacy deficiency, which included the following categories: avoidance, insolvency, interference from others, ineffective self-treatment, behavioral problems, stubbornness, superficiality, and denial.

**Conclusion:** It is of paramount importance to identify self-efficacy deficiency in families and promote problem- solving programs to increase family stability. In the present study, the main deficiencies in problem-solving were detected.

Family is a social foundation with various biological, economic, legal, psychological, and sociological aspects, forming the most fundamental social construction and the most basic social component reflecting the order or disorder in the community (1). Additionally, family is among the most significant factors affecting the community so that no society can enjoy health until it has healthy families (2).The results of studies in other countries indicated that family instability would lead to a wide spectrum of negative consequences including divorce, change in family structure, change in the economic status, and behavioral problems in children. 

On the other hand, family instability induces social instability, changes in social relations, and psychosocial incompatibility, etc. among the members of the family (3-6). Marital disputes, conflicts, and divorce are among the major problems in the Iranian community and are ranked among the 10 stressful life events (7). The statistical information on divorce in Iran indicates an increasing rate (8, 9). Many marriages fail after a short time, and thus understanding the causes can help find a solution to this problem (10). The families’ problem-solving ability increases stability in the family and reduces incompatibility and disputes among the couples.

This potency originates from the interventions based on a behavioral approach. The objective of the problem-solving method is to create a way for negotiation and find a logical solution to controversies (11).One of the problem-coping methods is problem-solving oriented coping method, which includes active problem-solving procedures in resolving stressful relationships between oneself and the environment (12, 13).The family’s ability in coping with problems, conflicts, and controversies suggests the familial efficacy to realize its roles, duties, and functions. On the other hand, any weakness in problem-solving ability may function as a potential factor in weakening the family foundation (14). A family’s self-efficacy deficiency in problem-solving is related to three sets of factors: (1) familial processes, the skills and abilities of the family in adapting to various conditions; (2) family content, income, occupation, psychosomatic health, and education level; and (3) familial social texture, values and beliefs of the family. Subsequently, any deficiency in the self-efficacy of any of the above-mentioned factors may lead to the incidence of some problems in family (15, 16). Moreover, low self-efficacy is characterized by coping strategies, such as denial and avoidance behavior, and manifests psychosomatic symptoms (17, 18). Based on the studies conducted, to date, on family stability and the consequences of family self-efficacy deficiency in solving familial problems, this issue should be considered very carefully and the related concepts should be analyzed. It seems necessary to conduct a qualitative study to identify the factors that play a significant role in demographic and cultural characteristics of couples. In this study, qualitative method was used, as we aimed at achieving the experiences of individuals and this was not possible using quantitative methods. A qualitative study will enable the researchers to perceive the real world of the individuals (19). Hence, we conducted a qualitative study to identify and describe the problem-solving methods running in families to be used as prompts for future research on stabilizing the foundation of the family in the country. To do so, we used directed content analysis based on the constructs of McMaster’s model, as problem- solving is one of the important domains of this model.

Studies that have used this model have shown that some families have many problems. For example, family function (such as problem solving) is impaired in families with a different cultural background (20), polygamous families (21), families with a member with a mental illness (22- 24), and those families with a member with physical problems and diseases (25, 26). On the other hand, McMaster's model is one of the most effective and suitable models for studying healthy families in Iran, which can be used by family counselors to prevent familial problems in the country (27).

Awareness about deficiency of self-efficacy related to problem- solving in families in Yazd (Iran) holds great potential for better understanding of this phenomenon. Identifying and understanding each of these aspects increases culturally suitable programs and service delivery. Although research has been done on families and their problems, these studies have not been based on a comprehensive model, especially in Yazd. In this qualitative study, we tried to consider cultural and social issues in Yazdi couples, and thus we aimed at detecting self-efficacy in problem-solving as a contributory factor in family instability in couples of Yazd (Iran), based on the McMaster model.

## Materials and Methods

*Sampling Strategy*

This qualitative study with content analysis was conducted as part of a multi-centered larger study on determining the experiences of family therapists, psychiatrists, and couples in Yazd, Iran. We used 2 heterogeneous samples to obtain the main themes and extract as many patterns as possible from different individuals and reach the best understanding of the phenomenon. We tried to describe family problem solving methods based on McMaster’s model using the directed content analysis in 2016. In this approach, analysis starts with a theory or a relevant research finding as a guide for initial codes. In this case, we decided to use directed content analysis because our research was theory- based (28). The goal of directed content analysis is to validate and develop the conceptual framework of a theory or expand the theory itself. Directed content analysis enjoys a more structured process than other content analysis methods (29, 30). 

McMaster's model was used in this study, as it is one of the effective models for investigating the function of families in Iran (27). The participants of the study included nine family therapists and psychiatrists, with a mean work experience of 14.2 years and four couples (eight persons) in Yazd, Iran, selected based on purposive sampling method. Considering the main goals of this study, the participants were selected among those who had enough experiences and power to express them. Strategies used to ensure the trustworthiness of this research were as follow: prolonged engagement (18 months), persistent observation, peer debriefing, member checking, and thick descriptions. All interviews were transcribed by a third party, who was blind to the aims of the study. Transcriptions were reviewed for accuracy by members of the research team, but not by the interviewers; and the necessary changes were made based on the audio files. All participants were given the opportunity to articulate their views and experiences during the interview process without being restricted by the topic guide. The researchers performed data collection, coding, and analysis at the same time. Constant comparison was applied in all processes of the analysis. Same codes were set in a same theme and were conceptualized. All codes were included in a reciprocating motion and revised if necessary. Moreover, all interviews were compared with each other. The interviews’ protocol was explained in data collection methods.

*Participants*

The participating couples were selected based on the goals of the study. Also, the family therapists and psychiatrists were selected based on their willingness to participate in the study and their experiences. The participants were selected among 9 family therapists and psychiatrists based on the following inclusion criteria: holding a PhD or MSc degree, having at least 5 years of work experience as a consultant, and inclination for participation. The couples were selected based on the following criteria: enjoying complete awareness, negative history of affliction with major psychological disorders, willingness to participate, having at least 1 child or more, and having complete consciousness (absence of history of insanity and dementia, lack of mental retardation, and awareness of time, place, and person). Moreover, we increased the reliability to reduce errors by providing a deep description, recording all the work done in the research process from the start to the end, interviewing with different persons, and conducting interviews in a quiet place. 

*Data Collection and Study Methods*

Data were collected using semi-structured and deep interview. The basis of this research was localization of McMaster's model for the population of Yazd (Iran). This article was a part of a broad research that expanded the model with new domains in the main research. McMaster model of family functioning, offered in the early 1960s at McMaster University by Epstein, Bishop, and Levin, provided a comprehensive approach in the field of family therapy by combining the strengths of all previous models. McMaster model evaluates marriages and families. This model is based on a system theory and describes structure, organization, and exchange pattern of the marital unit, allowing family or marital relationships to be tested on a surface spectrum, from health to severe mental disorder (25).The questionnaire related to this model (Family Assessment Device) contains 60 questions and evaluates 6 dimensions including problem solving, communication, roles, emotional responses, emotional involvement, behavioral control, and general function (31, 32).

The data collection instrument was voice recorder, which was then audio-recorded and transcribed verbatim. We started with a joint interview to increase participants’ ease and confidence during the interview. The main question of individual open interviews was as follows: “Please tell us about your experiences in your usual day in the family.” Then, to clarify the concepts pertaining to problem-solving and to deepen the interview, some questions were asked, which are as follow: “What is your experience about different ways of problem- solving in your family?” and “What is the barrier of reducing efficacy in problem-solving in your family?” After the required permission was obtained, all the interviews were recorded, completely transcribed at the end of each interview, and analyzed using directed content analysis. The average duration of the interviews was 45 to 60 minutes and was performed with appointments made beforehand at the family therapists and psychiatrists’ workplace or in a place determined by the couples, where they felt comfortable. The control mechanisms of data saturation were as follow: constant comparison of codes and distinguishing the differences and similarities between the initial codes, revising some codes if necessary, long-term involvement with the phenomenon (about 18 months), sequential interviews, reviewing the findings with participants, and overview of observers. When no new data could be extracted from the participants, we stopped data collection. Furthermore, members and the expert panel checked data to ensure data saturation.

*Data Analysis*

In accordance with the aims of the study, the analysis was focused on couples’ problem- solving strategies in their families. This study used qualitative directed content analysis, which included the following steps: (1) transcribing the whole interview immediately after its completion; (2) reviewing the whole text for arriving at a general understanding of its content; (3) determining the semantic units and primary codes; (4) classifying similar primary codes in more comprehensive categories; and (5) determining the main theme of the categories (33).Therefore, ZP started the analysis by checking the transcripts of the interviews to ensure accuracy (34). Each transcript was read several times to identify initial patterns (35). The transcripts were read carefully line-by-line, and initial codes were developed (35, 36).The coding process was as follow: comparing the data, asking questions, and reviewing notes. After open coding, an initial coding scheme was developed that guided the coding of the remaining transcripts. In the process, codes were repeatedly modified or combined, and parts of transcripts were recoded. Codes were then sorted into emerging categories based on relations and interlinks. These categories were further combined into hierarchical structures, if possible (37). Data from the field notes were used to further inform the development of codes and categories. To increase the rigor of the analysis, the fourth and sixth authors (HT and AAV) analyzed the transcripts independently and combined the results with the first author. Coding validation was done by original data availability (participants); clarifying coding, evidence based writing, stepwise replication, external reviewer, and debriefing.

All identifying information (names, locations, identifiable information, etc.) was removed from quotes to ensure anonymity, and pseudonyms were used. The reliability and validity of the data were established using sufficient participation, close interaction with the participants, variety in the participants in age, gender, work experience, etc., data integration, repeated review of data, data review by the participants, and ethic perspectives of outsider observers. Also, we selected couples from different socioeconomic areas. Besides an individual analysis, we performed a total analysis by combining interviews, which was difficult and time-consuming, but increased external validity.

*Ethical Considerations*

The researcher obtained the approval of the Institutional Review Board and the Research Ethics Committee of Health School of Shahid Sadoughi University of Medical Sciences (Ethic code: IR.SSU.SPH.REC.1395 .52) Yazd, Iran. All participants in the study were assured of the confidentiality of their personal information and absence of any constraint to participate in the study.

## Results

We analyzed 17semi-structured interviews that were conducted with nine family therapists and psychiatrists (FT&P) and four couples (E) in Yazd, Iran (Table 1).

On the whole, 17 interviews with the participants resulted in 185 codes without calculating the overlaps. By considering the overlaps and integrating them for more accurate codes, 44 codes remained, and because they could not be in any of the categories, some of them were placed in subsequent interviews. The process of comparative analysis continued till all the similar codes were compared based on similarity, correspondence, and suitability. Similar cases were written as one code or category. Ultimately, eight main categories were obtained. The categories were based on the opinions of the experts. The main categories of avoidance, insolvency, interference from others, ineffective self-treatment, behavioral problems, stubbornness, superficiality, and denial were conceptualized as the main categories. Table 2 presents the categories, which are as follow: 

*1. Avoidance*

One of the main categories obtained was avoidance. Avoiding the problem has led to self-efficacy deficiency in problem-solving in some cases. One of the wives said, “At the time of dispute, I just leave the situation saying nothing, but then our children interfere and everything will be fine again. I never leave home.” 

*2. Insolvency*

Insolvency occurred in many cases due to lack of change in the behavior of the couples, their inability to solve the disputes and disparities, and their obligatory adaptation. An expert participant stated, 

“Anyhow, there are individuals who get involved in insolvency and distress, and they say they don’t know what else to do, as everything they did was not enough to solve the problem.”

*3. Interference from others*

One of families’ weaknesses in self-efficacy in problem-solving is interference of others, specifically from the couples’ parents. This is more marked at the first years of marriage. These interferences can detrimentally exert some negative effects on the relationships between the couples. The experiences of the couples in this regard are as follow:

 “The couple’s parents play a basic role. If they interfere unnecessarily, it will have a negative effect. The couples transmit their problems to the parents and the parents provide strategies frequently. This causes the disintegration of families. I think extreme relationships are not good, I mean parents have no duties after children are married.”

*4. Ineffective self-treatment*

Ineffective self-treatment includes the use of herbal medicines and consumption of chemical drugs. This problem is more prevalent in traditional families with lower education. One expert states, “The clients first express their problems to the friends and relatives and very often use herbal medicines or get it directly from the pharmacy and if it was useless, then they would turn to us.”

*5. Behavioral Problems*

According to the participants’ experiences, physical beating, insulting, scolding, disputing, and lack of respect for adults were among the major manifestations of behavioral problems. As one expert noted, “There are some conflicts occasionally. Most of the times, parents complain of violence and disobedience on the part of their offspring, especially during teen years. Sometimes, parents react to them by psychological reactions, for example, they display violence instead of solving the problem.”

*6. Stubbornness*

Stubbornness and insistence on one’s position in the familial life can function as a basic significant factor in the conflict between the couples. In this study, the husband’s insistence on his position was noticeable. Below is the experience of one of the participants: 

 “In the cases I face, I try to talk and utter my inner beliefs, especially if I am right, but this annoys my wife.” 

*7. Superficiality*

Superficiality indicates that the couples do not pay attention to major problems, and instead of finding a proper single solution, they apply all the possible solutions and try them one by one. An expert states, “Most couples don’t adopt a useful method to prevent the problems. The families who refer to us are those who have tried every possible solution and did, in their own opinion, the right thing without any obvious results. They did not try to see where the major problem was.”

*8. Denial*

Denial is one of the defensive mechanisms against problems. Based on the participants’ assertions, lack of acceptance of the problem on the part of the parents and rendering the problems, especially with respect to children, are barriers against the acceptance of the problem. One participant said, “The main point is the perception of the problem itself. Families feel that the problem is something natural and they do not need the help of a professional. When they fail to resolve it, they understand that there is a problem and they have to seek professional help.”

**Table1:** The Demographic Characteristics of the Participants (n = 17)

**Code**	**Gender**	**Age**	**Education level**	**Work Experience (year)**
FT&P _1_ FT&P _2_ FT&P _3_ FT&P _4_ FT&P _5_ FT&P _6_ FT&P _7_ FT&P _8_ FT&P _9_	Male Male Male Male Male Female Female Female Female	56 48 53 36 43 49 52 38 40	Specialist Specialist MS_C_ Specialist Specialist MS_C_ Specialist Specialist MS_C_	21 17 18 9 11 15 17 8 12
C_1_ C_2_ C_3_ C_4_ C_5_ C_6_ C_7_ C_8_	Male Female Male Female Male Female Male Female	51 47 35 28 58 53 47 40	Diploma Diploma PhD Bachelor Diploma Diploma Bachelor Diploma	

FT&P: Family Therapists and Psychiatrists; C: Couples

**Table2:** Self-efficacy Deficiency in Problem-solving Among Couples in Yazd (Iran)

**Theme**	**Categories**	**Subcategories**
Deficiency of self-efficacy	Avoidance	-Sulking -Ignoring each other -Withdrawing -Leaving home
	Insolvency	-Adaptation due to lack of change in the opposite partner’s behavior -Acceptance of family problems -Forgiving of disputes and controversies -Obligatory colluding interaction
	Interference from others	-Interference from the espouses’ parents -Interference from relatives -Interference from friends
	Ineffective self-treatment	-Use of traditional medicine -Resorting to herbal medicines -Use of chemical medicines as self-treatment
	Behavioral problems	-Insulting and scolding -Disrespecting others -Physical violation and beating
	Stubbornness	-Lack of apology -Imposing one’s want on others -Lack of forgiveness
	Superficiality	-Lack of investigation of the main problem -Use of all possible solutions -Lack of use of appropriate solutions
	Denial	-Lack of acceptance of problems on the part of the husband -Lack of acceptance of problems on the part of the wife -Considering problems as natural issues

The researchers carried outperformed data collection, coding and analyzing data at the same time. Constant comparison was applied in all processes of the analysis, and the differences and similarities between the initial codes were distinguished. Same codes were set in same categories. All codes were included in a reciprocating motion and they revised if it was necessary. Moreover, all interviews were compared with each other. Finally, we identified eight main categories and 26 subcategories in participants’ responses about self-efficacy deficiency in problem-solving among the couples using purposive sampling method. From 44 codes that could not be in any of the categories, some of them were placed in subsequent interviews and 6 codes were omitted because one code could not be classified in a categotry (Table 2).

## Discussion

Problem-solving is one of the techniques found in cognitive-behavioral approaches. In this procedure, family members are helped to use their abilities and qualifications in coping with their daily living problems. Indeed, individuals should select the most useful and most practical solution. They should also utilize the selected solution effectively (38). Problem- solving, the first dimension of the McMaster Family Function (MMFF), refers to the family’s ability to resolve problems that threaten the integrity and functional capacity of the family. Items of this dimension include decision- making about the problems, discussing the efforts of the family to solve problems, resolving emotional disappointments, confronting problems involving feelings, and trying to think of different ways to solve problems (39).

Some studies suggested that high self-efficacy is often correlated with problem-centered coping strategies. When faced with stressful situations, persons with higher self-efficacy who have some control on their thoughts display greater stability. High self-efficacy is associated with problem-solving (40-42)

One aspect of marital adaptation is the ability for conflict resolution and exposure to problems. The existence of conflict in marital relations is inevitable. The important point is their ability to face the conflict and its resolution. The couple should not be afraid of problems and should not avoid them (43). Another variable of the family system is insolvency. Insolvent persons think that the events are out of their control. Insolvency may be attributed to low levels of control on situations. The amount of insolvency is not significantly different between men and women (44). Furthermore, with regards to interference from others, Vakili et al. (2006) cited in their study that interference from others ranks as one of the most important reasons of disputes (16.8%) in families from the couples’ viewpoint. (45). Based on the data collected during 1997 and 1980 on the causes of divorce, Amato &Previti (2003) stated that from the respondents’ point of view, 2.4% of the causes of divorce is related to interference from the family and relatives as external factors (46). Davoodi et al. (2012, cited in Conway, 2002) indicated that marital compatibility is not produced automatically; rather, it demands some efforts by the couple. Helping the couple to find solutions to their problems, develop suitable solutions instead of ineffective self-treatment, and mark the exceptions in life may affect the marital compatibility and strengthening of the family (47). Hence, the familial stability is jeopardized by superficiality, lack of investigation of the major problems, the use of all available solutions, and inaccessibility of the suitable and effective solutions to problems.

Additionally, with increase in age, stubbornness and its related behaviors are manifested more vividly. Heid et al. (2016) stated that 77% of children and 66% of parents reported parents’ stubbornness (48). In this respect, Guttman&Krokoff (1989) stated that defensive approach, stubbornness, and lack of interaction disturb marital relations (49). Furthermore, Villant (1994) emphasized that defensive mechanisms are unconscious internal mental processes that protect the self against threatening or stressful situations; yet, the unsuitable use of these mechanisms like denial may impair mental development and hinder the proper coping responses (50). In this study, quarrel was an ineffective way of problem-solving to resolve behavioral problems between the couples. Quarrel is a phenomenon, which destabilizes healthy relationships between family members, and it is an increasing worldwide problem threatening the mental health of women and children. The data available demonstrate that 20% to 50% of women in most countries experience violence and quarrel at some stage in their lives (51, 52). It seems that under certain circumstances including interference from others, self-treatment, avoiding the problem, insolvency, stubbornness, superficiality, denial, and behavioral problems, specifically, quarrel and violence, the families’ ability to control life affairs decreases, leading to a reduction in families’ self-efficacy for problem-solving. This culminates in weakness in life management, ignoring personal needs, and indifference about the family’s psychosomatic health. Devoting some time to discuss the problems, allocating the share of each family member, listing the possible solutions, agreeing on the best possible solution, and removing conflict are among the issues that the families should be trained for to be able to find logical solutions to their familial problems. The important role of problem- solving strategies in family stability encouraged us to conduct this study in Yazd. In our study, participants agreed that their self-efficacy deficiency in problem- solving could negatively affect family function and its stability. Thus, families should acquire problem-solving skills to reduce the odds of incidence of detrimental consequences due to their self-efficacy deficiency in problem-solving. Our findings suggest possible strategies for encouraging couples to support each other in pursuing their own preferred strategies of problem- solving. Our results can be used by family counselors to help prevent familial problems in our country and have implications for researchers, educators, family therapists and psychiatrists. 

## Limitations

Family therapists and psychiatrists in the study were selected based on their willingness to participate in the study. Therefore, it is possible that those who chose to participate in the study were those who were more enthusiastic and committed to the study. It was not possible to interview all the experts due to unwillingness of some of them. This limitation was resolved as much as possible by motivating them in various ways. Future qualitative and quantitative research with larger sample sizes should be conducted separately to obtain a precise perspective about problem- solving strategies in families. 

## Conclusion

The problem-solving ability is one of the important factors contributing to family stability. Based on our findings, the families in Yazd (Iran) made some attempts to solve their problems but they were not successful, and they suffered from self-efficacy deficiency including avoidance, insolvency, "interference from others, ineffective self-treatment, behavioral problems, stubbornness, superficiality, and denial in their problem-solving strategies. Thus, it is necessary to identify the barriers of self-efficacy in families and promote problem-solving programs in Iran to increase family stability. In the present study, due to the presence of self-efficacy deficiency in problem-solving, the main deficiencies were obtained.
